# NAC1 modulates autoimmunity by suppressing regulatory T cell–mediated tolerance

**DOI:** 10.1126/sciadv.abo0183

**Published:** 2022-06-29

**Authors:** Jin-Ming Yang, Yijie Ren, Anil Kumar, Xiaofang Xiong, Jugal Kishore Das, Hao-Yun Peng, Liqing Wang, Xingcong Ren, Yi Zhang, Cheng Ji, Yan Cheng, Li Zhang, Robert C. Alaniz, Paul de Figueiredo, Deyu Fang, Hongwei Zhou, Xiaoqi Liu, Jianlong Wang, Jianxun Song

**Affiliations:** 1 Department of Toxicology and Cancer Biology, Department of Pharmacology and Nutritional Science, and Markey Cancer Center, University of Kentucky College of Medicine, Lexington, KY 40536, USA.; 2 Department of Microbial Pathogenesis and Immunology, Texas A&M University Health Science Center, Bryan, TX 77807, USA.; 3 Department of Veterinary Pathobiology, Texas A&M University, College Station, TX 77845, USA.; 4 Norman Borlaug Center, Texas A&M University, College Station, TX 77845, USA.; 5 Department of Pathology, Northwestern University Feinberg School of Medicine, Chicago, IL 60611, USA.; 6 Department of Medicine, Columbia Center for Human Development, Columbia University Irving Medical Center, New York, NY 10032, USA.

## Abstract

We report here that nucleus accumbens–associated protein-1 (NAC1), a nuclear factor of the Broad-complex, Tramtrack, Bric-a-brac/poxvirus and zinc finger (BTB/POZ) gene family, is a negative regulator of FoxP3 in regulatory T cells (T_regs_) and a critical determinant of immune tolerance. Phenotypically, NAC1^−/−^ mice showed substantial tolerance to the induction of autoimmunity and generated a larger amount of CD4^+^ T_regs_ that exhibit a higher metabolic profile and immune-suppressive activity, increased acetylation and expression of FoxP3, and slower turnover of this transcription factor. Treatment of T_regs_ with the proinflammatory cytokines interleukin-1β or tumor necrosis factor–α induced a robust up-regulation of NAC1 but evident down-regulation of FoxP3 as well as the acetylated FoxP3. These findings imply that NAC1 acts as a trigger of the immune response through destabilization of T_regs_ and suppression of tolerance induction, and targeting of NAC1 warrants further exploration as a potential tolerogenic strategy for treatment of autoimmune disorders.

## Teaser

NAC1, a POZ/BTB protein, modulates autoimmunity by suppressing regulatory T cell–mediated tolerance.

## INTRODUCTION

Aberrant autoimmunity results in over 80 different autoimmune diseases that are often debilitating and life-threatening, for which there is no cure at present. Autoimmune diseases such as Crohn’s disease, type 1 diabetes mellitus, rheumatoid arthritis, and ulcerative colitis are believed to result from interaction between genetic and environmental factors and to be a consequence of compromised immune tolerance versus adaptive immune response. Immune tolerance prevents an immune response to a particular antigen (Ag) or tissues that cause autoimmune disorders, and a range of immune cell types participate in the control of hyposensitivity of the adaptive immune system to the self-Ag or non–self-Ag.

Among these immune cells, FoxP3^+^ regulatory T cells (T_regs_), a distinct and dynamic subset of CD4^+^ T cells, are an essential contributor to the immune tolerance, maintenance of immune cell homeostasis, and the balance of the immune system, and defects in T_regs_ occur in virtually all the autoimmune disorders (*1*). The stability of the suppressor T_regs_ is critical for their function but is reduced in most of the autoimmune disorders. Therefore, maintenance of the T_reg_ stability is crucial for immunologic tolerance. However, how impaired balance between immune response and tolerance is triggered and the key molecular determinants that affect T_reg_ stability remain elusive.

Here, we report our new finding that nucleus accumbens–associated protein-1 (NAC1), encoded by the *NACC1* gene and originally identified as a cocaine-inducible transcript from the nucleus accumbens (*2*), acts as a vital modulator of immune suppression. NAC1 is a nuclear factor that belongs to the BTB (Broad-complex, Tramtrack, and Bric-a-brac)/POZ (poxvirus and zinc finger) gene family. Using NAC1-deficient (^−/−^) mice, we uncovered a previously unrecognized but important role of NAC1 in triggering autoimmunity and T_reg_ instability and demonstrated that NAC1 contributes to the break of immune tolerance through its negative control of T_reg_ development and function associated with deacetylation and destabilization of FoxP3 protein.

## RESULTS

### Overall T cell population is divergent in the wild-type and NAC1^−/−^ mice

**Fig. 1.**
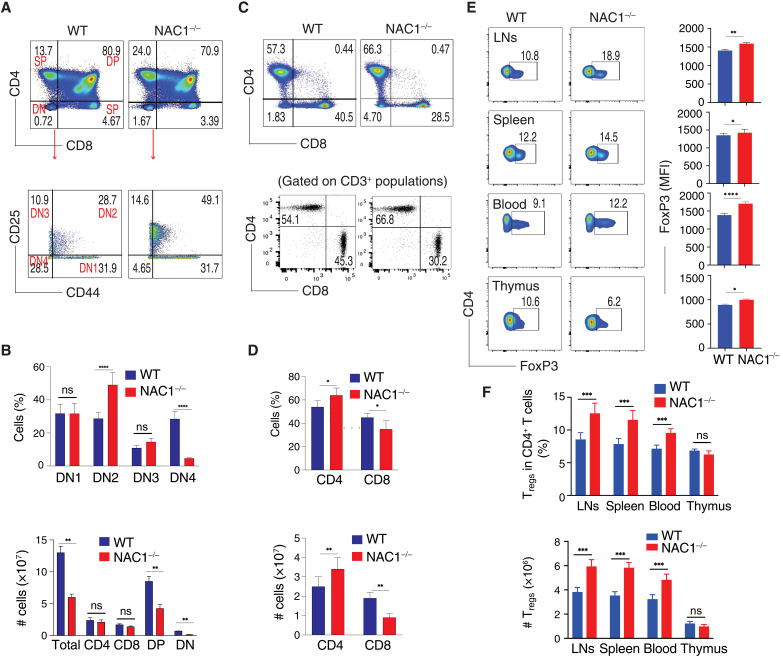
Loss of NAC1 affects overall T cell populations. T cells from the thymus, peripheral LNs, and spleen of WT or NAC1^−/−^ mice were analyzed by flow cytometry and calculated for numbers or percentages. (**A**) CD4 and CD8 in the thymus. The DN populations were analyzed for DN1 to DN4 stages on the basis of CD44 and CD25. Data shown are the representative of five mice per group of three independent experiments. (**B**) Numbers and percentages of total thymocytes, CD4 or CD8 SP, and the percentages of DN2 or DN4 cells. Data shown are the representative of three identical experiments. The values represent means ± SD (*N* = 4 or 5). **P < 0.01, ****P < 0.0001, ns, no difference, Student’s unpaired *t* test. (**C**) CD4 and CD8 T cells from the pooled LNs and spleen. Data shown are the representative of five mice per group of three independent experiments. (**D**) Numbers and percentages of T cells from the pooled LNs and spleen. *P < 0.05, **P < 0.01. (**E**) Representative CD4^+^FoxP3^+^ T_regs_ in the pooled LNs, spleen, blood, and thymus, gating on CD4^+^ populations. Data shown are the representative of three identical experiments (*N* = 4 or 5). *P < 0.05, **P < 0.01, ****P < 0.0001. (**F**) Numbers and percentages of T_regs_. Data shown are the representative of three identical experiments. The values represent the means ± SD (*N* = 4 or 5). ***P < 0.001; ns, no statistical difference, Student’s unpaired *t* test.

Previous studies have shown that NAC1 participates in the regulation of the self-renewal and pluripotency of embryonic stem cells (*3*–*5*) and somatic cell reprogramming (*6*), and we recently found that NAC1 has a critical role in cellular metabolism (*7*). As metabolic reprogramming can considerably influence T cell activation, expansion, and effector function (*8*, *9*), we queried whether NAC1 affects T cell development and function. We first performed T cell profiling in wild-type (WT) and NAC1^−/−^ mice. Compared with WT mice, development of T cells in the thymus of NAC1^−/−^ mice was curbed, as evidenced by increased numbers of thymocytes in the dominant-negative (DN) stage (1.67 versus 0.72%) and decreased numbers of cells in the DN4 stage (4.65 versus 28.5%; *P* < 0.0001; Fig. 1A). Although NAC1^−/−^ mice showed a reduction of total thymocytes and decreased cell number in the DN4 stage, an accumulation of T cells in the DN2 stage was observed in those animals (49.1% versus 28.7%; *P* < 0.0001; Fig. 1, A and B). These alterations were correlated with a decreased percentage of TCRβ^+^ cells in DN4 cells found in NAC1^−/−^ mice. Despite a higher percentage of DN cells in NAC1^−/−^ mice, which show higher numbers of CD117^+^ cells, WT animals had a greater number of TCRβ^+^ cells than NAC1^−/−^ mice. Like the gating on the DN4 population, there was a decrease in TCRβ^+^ cells in NAC1^−/−^ mice as compared with WT animals, which could be due to a higher percentage of DN4 cells in WT mice (fig. S1). Conversely, in the lymph nodes (LNs) and spleen of NAC1^−/−^ mice, there was a reduced percentage of CD8^+^ T cells (28.5 versus 40.5%) but an increased percentage of CD4^+^ T cells (66.3 versus 57.3%) as compared with the controls (Fig. 1C). Despite the similar numbers of the total CD4^+^ or CD8^+^ single-positive (SP) T cells in the thymus (Fig. 1B; bottom), there were significant increases in percentage and numbers of CD4^+^ SP T cells (*P* < 0.05 or *P* < 0.01) but a decrease in CD8^+^ SP T cells (*P* < 0.05 or *P* < 0.01) in the pooled LNs and spleen in NAC1^−/−^ mice, as compared with WT controls (Fig. 1D, lower panel). These results suggest an important role for NAC1 in the early stage of T cell development and in the differentiation of CD4^+^ and CD8^+^ SP cells.

### Deficiency of NAC1 promotes T_reg_ development and stability

**Fig. 2.**
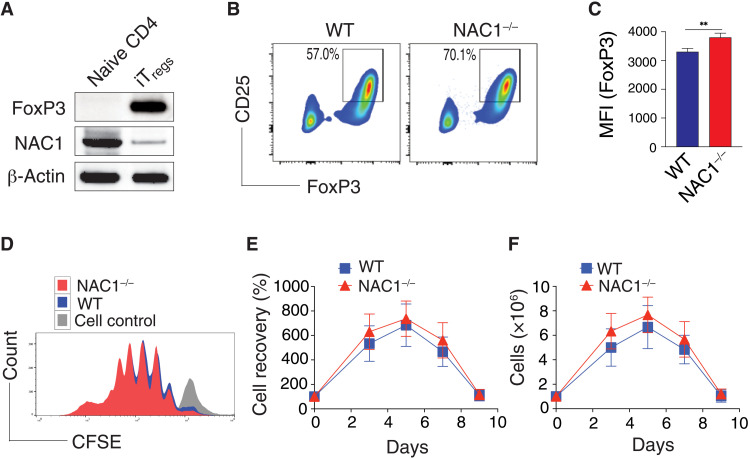
Loss of NAC1 enhances the induction of iT_regs_ but not cell division and survival. The naive CD4^+^CD25^−^ T cells from the pooled spleen and LNs of WT or NAC1^−/−^ mice were induced to iT_regs_ in vitro in the presence of TGF-β for 5 days (**A** to **C**), or purified CD4^+^ T_regs_ from the pooled LNs and spleen of WT or NAC1^−/−^ mice were stimulated with anti-CD3 plus CD28 antibodies in the presence of rIL-2 for various times (**D** to **F**). (A) Expression of NAC1 in naïve CD4 and iT_regs_ of WT T cells detected by immunoblots. (B) Expression of CD25 and FoxP3 in iT_regs_ generated from WT and NAC1^−/−^ CD4^+^CD25^−^ T cells by flow cytometry. Data shown are the representative of three identical experiments. (C) MFI of FoxP3 in iT_regs_ generated from WT and NAC1^−/−^ CD4^+^CD25^−^ T cells as analyzed by flow cytometry. Results shown are the means ± SD of three identical experiments. **P < 0.01, Student’s unpaired *t* test. (D) Cell proliferation/division by carboxyfluorescein diacetate succinimidyl ester (CFSE)–based flow cytometry. Data shown are the representative of three identical experiments. (E) Percentage and (F) numbers of cell recovery on various days were examined by trypan blue exclusion. The numbers of T cells present on day 0 were assigned a value of 100%, and numbers surviving on various days were used to calculate the percentage recovery relative to day 0. Data shown represent the means ± SEM of percentage change or numbers of three independent experiments. All *P* > 0.05, Student’s unpaired *t* test.

The significant increase in total peripheral CD4^+^ T cell population observed in NAC1^−/−^ mice (Fig. 1D) prompted us to ask whether NAC1 plays a regulatory role in the development of T_regs_, a unique subtype of CD4^+^ T cells able to suppress excessive immune reaction. As compared with WT animals, NAC1^−/−^ mice showed an evident increase in percentage of T_regs_ in the LNs, spleen, and blood (Fig. 1E). Moreover, NAC1^−/−^ animals had significantly higher percentage and numbers of T_regs_ in the LNs and spleen but not in the thymus (*P* < 0.0001; Fig. 1F). Although NAC1 deficiency led to reduced frequencies of T_regs_ (10.6% in WT and 6.2% in NAC1^−/−^; Fig. 1F; *P* > 0.05), the FoxP3 expression [mean fluorescence intensity (MFI)] was significantly higher in NAC1^−/−^ T_regs_ than in the WT control (Fig. 1E; *P* < 0.05). To prove the role of NAC1 in the development of T_regs_, we used an in vitro system in which induced T_regs_ (iT_regs_) are generated from naive CD4^+^CD25^−^ T cells (*10*). The naive CD4^+^CD25^−^ T cells from the LNs and spleen of WT or NAC1^−/−^ mice were treated with transforming growth factor–β (TGF-β) and interleukin-2 (IL-2) to produce iT_regs_. The naive CD4^+^CD25^−^ T cells from WT mice expressed abundant NAC1 but no detectable FoxP3; notably, the iT_regs_ from those T cells showed a robust expression of FoxP3 but a substantial reduction of NAC1 expression (Fig. 2A). A significantly greater amount of iT_regs_ were generated from NAC1^−/−^ than from WT CD4^+^CD25^−^ T cells (Fig. 2, B and C). Furthermore, T_regs_ from WT or NAC1^−/−^ mice had similar proliferation and survival profiles (Fig. 2, D to F). These results suggest that NAC1 may have a role in regulating T_reg_ development at early stages in the thymus. In addition to affecting CD4^+^ populations, NAC1 also affects CD8^+^ T cells. NAC1 deficiency led to a significant decrease in CD8^+^ SP T cell generation in the LNs and spleen, reduced production of cytokine, and shortened cellular survival (fig. S2).

### NAC1^−/−^ T_regs_ display enhanced functional activities

**Fig. 3.**
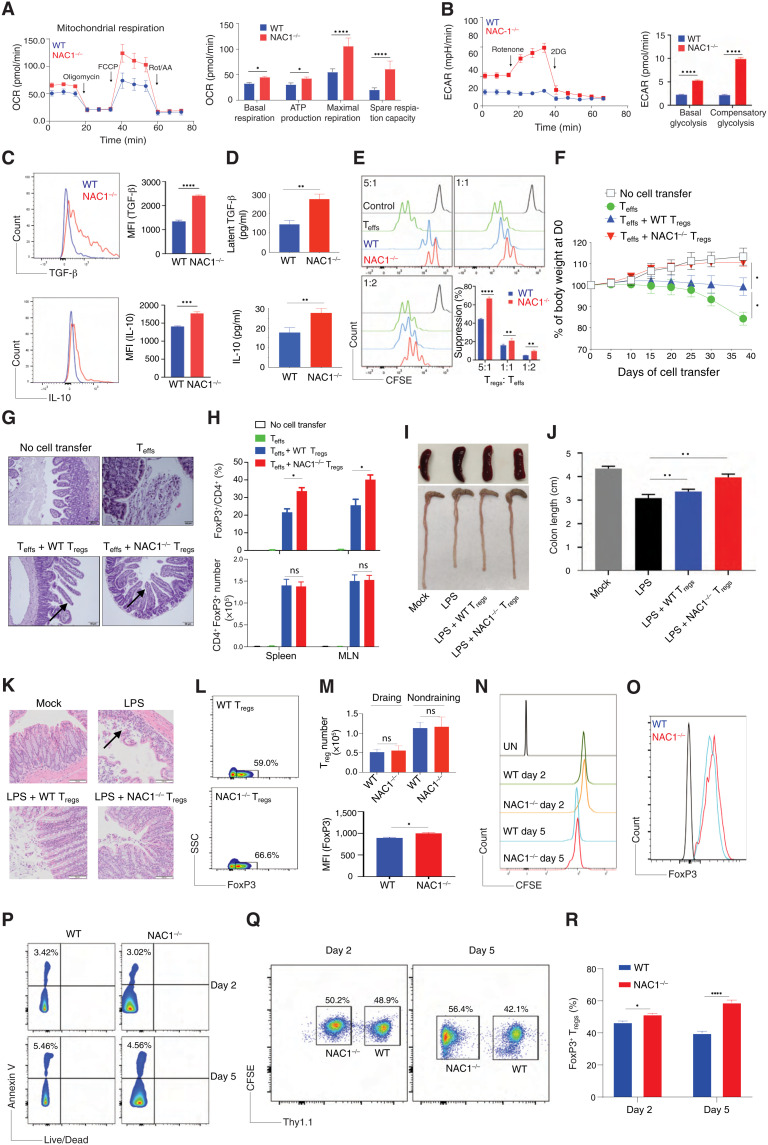
Loss of NAC1 enhances the functional activity of T_regs_. Purified CD4^+^ T_regs_ from the pooled LNs and spleen of WT or NAC1^−/−^ mice were stimulated with anti-CD3 plus CD28 antibodies in the presence of rIL-2 for various times. (**A**) OCR. (**B**) ECAR. (**C**) Cytokine production by intracellular staining. (**D**) Cytokine secretion by ELISA. (**E**) In vitro suppressive assay. (**F** to **I**) T cell transfer model of colitis. (F) Changes of body weight. (G) Representative hematoxylin and eosin (H&E)–stained sections of gut tissues. (H) T_reg_ frequencies/numbers in the adoptive transfer–induced colitis model. (I and **J** to **M**) Lipopolysaccharide (LPS)–mediated colitis. (I) Representative colon images of the LPS-induced colitis. (J) Colon lengths in LPS-induced colitis. (K) Representative H&E-stained sections of images of proximal colon cross section. (L and M) Numbers of transferred T_regs_ and FoxP3 MFI (bottom), gating on Thy1.2^+^ populations (top). (**N** to **P**) In vivo analyses of T_regs_. WT and NAC1^−/−^ T_regs_ (Thy1.2^+^) were labeled with CFSE and intravenously injected into mice. On days 2 and 5, the transferred Th1.2^+^ T_regs_ were analyzed by flow cytometry. (N) Proliferation by CFSE. (M) FoxP3 expression. (P) Apoptosis. (**Q** and **R**) In vivo cotransfer of CFSE-labeled WT (Thy1.1^+^) and NAC1^−/−^ (Thy1.2^+^) at 1:1 ratio into recipients and examined the NAC1^−/−^/WT ratios of T_regs_ on days 2 and 5. Data shown are the means ± SD of three independent experiments and are the representative of three identical experiments. *P < 0.05, **P < 0.01, ***P < 0.001, ****P < 0.0001, Student’s unpaired *t* test.

To further validate the negative control of T_reg_ development by NAC1, we examined the functional activity of the T_regs_ either from WT or NAC1^−/−^ mice. CD4^+^CD25^+^ T_regs_ from the LNs and spleen of WT or NAC1^−/−^ mice were stimulated with the mouse CD3/CD28-loaded beads in the presence of rIL-2, and the metabolic differences between WT and NAC1^−/−^ cells were then analyzed using the Seahorse XF Cell Mito Stress Test Kit. Although T_regs_ from WT or NAC1^−/−^ mice had similar proliferation and survival profiles (Fig. 2, D to F), T_regs_ from NAC1^−/−^ mice exhibited a significantly higher oxygen consumption rate (OCR) and extracellular acidification rate (ECAR) than T_regs_ from WT mice (Fig. 3, A and B), suggesting that NAC1^−/−^ T_regs_ are metabolically more active than the corresponding control T_regs_. Consistently, NAC1^−/−^ T_regs_ produced significantly greater amounts of the suppressive cytokines, TGF-β and IL-10, than WT T_regs_, as shown by intracellular staining (Fig. 3C) and enzyme-linked immunosorbent assay (ELISA; Fig. 3D), which may constitute one of several mechanisms that contribute to the suppressive capacity of T_regs_. These results clearly demonstrate a negative impact of NAC1 on the suppressive function of T_regs_ and imply that inhibiting NAC1 may modulate autoimmunity through promoting T_reg_ development and function. The enhanced function of NAC1^−/−^ T_regs_ was further demonstrated in an in vitro suppressive assay (Fig. 3E) and in an autoimmune colitis model subjected to in vivo cotransfer of T_regs_ with CD4^+^ T effectors (T_effs_), which showed that NAC1^−/−^ T_regs_ elicited a stronger immune-suppressive effect on inflammation than WT T_regs_. Transfer of naive T_effs_ into *Rag*1^−/−^ mice resulted in weight loss in 2 weeks, and cotransfer of T_effs_ with FoxP3^+^ T_regs_ (T_reg_/T_effs_ ratio of 1:3) from NAC1^−/−^ mice led to a significant reduction of weight loss compared with those of WT mice (*P* < 0.05; Fig. 3F), which was associated with increased T_reg_ frequencies but not numbers in the spleen and mesenteric LNs (Fig. 3H). The cotransfer of T_effs_ with NAC1^−/−^ and WT FoxP3^+^ T_regs_ effectively protected from a decrease in finger-like villus projections (arrows) in the gut (Fig. 3G). Similarly, in the lipopolysaccharide (LPS)–induced colitis, adoptive transfer of NAC1^−/−^ T_regs_ was more efficient in preventing colon loss than WT T_regs_, as shown by the colon lengths (Fig. 3, I and J), histology (an arrow indicates the goblet cell architecture of the colon tissue was damaged by LPS; Fig. 3K), and T_reg_ infiltration in the draining LNs (Fig. 3, L and M). Furthermore, we performed in vivo analyses on survival, proliferation/cell cycle, and apoptosis. WT and NAC1^−/−^ T_regs_ (Thy1.2^+^) were labeled with carboxyfluorescein diacetate succinimidyl ester (CFSE) and intravenously injected into B6.Thy1.1 Tg mice. On days 2 and 5 after T_reg_ transfer, the pooled LNs and spleen were analyzed for the transferred Th1.2^+^ T_regs_ using flow cytometry. We found that NAC1^−/−^ T_regs_ had similar survival and proliferation/cell cycle (Fig. 3N) but increased FoxP3 expression (Fig. 3O) and reduced apoptosis (Fig. 3P) as compared with WT T_regs_. Moreover, we performed the experiment in vivo cotransfer study of CFSE-labeled WT (Thy1.1^+^) and NAC1^−/−^ (Thy1.2^+^) at 1:1 ratio into Thy1.1 recipient and examined the NAC1^−/−^/WT T_reg_ ratio on days 2 and 5. We found that the ratio changed from 50.2%/48.9% = 1.0 to 56.4%/42.1% = 1.3 (Fig. 3, Q and R). Together, in the peripheral tissue, that reduced apoptosis and increased T_reg_ stabilization through up-regulation of FoxP3 expression in NAC1^−/−^ T_regs_ are accounted for enhanced T_reg_ frequency. In addition, we found that both NAC1^−/−^ iT_regs_ and natural T_regs_ (nT_regs_) produced more suppressive cytokines (i.e., IL-10 and TGF-β) than WT cells, and the similarity between iT_regs_ and nT_regs_ (fig. S3). In a mouse tumor model receiving the in vivo cotransfer of T_regs_ with CD8^+^ T cells, NAC1^−/−^ T_regs_ displayed greater suppressive effect on antitumor immunity than the control T_regs_ (fig. S4), another evidence for the enhanced suppressive activity of NAC1^−/−^ T_regs_.

### NAC1^−/−^ mice are insusceptible to induction of autoimmunity

**Fig. 4.**
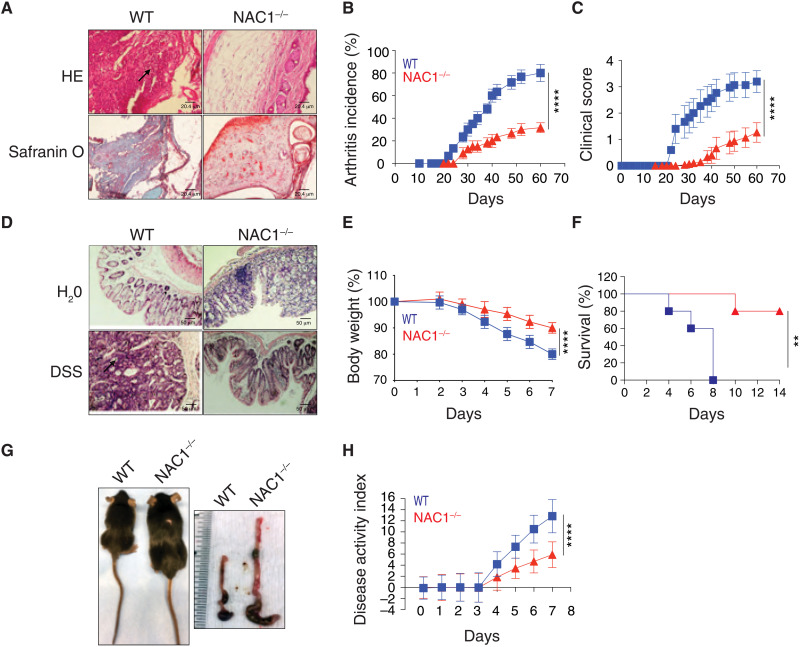
NAC1^−/−^ mice are tolerant to induction of autoimmunity. WT or NAC1^−/−^ mice were challenged with either bovine type II collagen in complete Freund’s adjuvant by one intradermal immunization at two sites in the base and slightly above of the tail on day 0, or by oral ingestion of 3% DSS (MP Biomedicals) in drinking water for 5 days. (**A** to **C**) Arthritis: The histology of the joints (A), arthritis incidence (B), and clinical score (C) were evaluated by examining the paws. Values are the means ± SEM of three independent experiments (*n* = 10). ****P < 0.0001 in (B) and (C), simple linear regression. (**D** to **H**) Colitis: The severity of colitis activity was graded on the designated dates. Histology of colon (D), animal body weight change (E), survival (F), animal size and colon length (G), and the resultant IBD disease activity index (H) were determined. Values are the means ± SEM of three independent experiments (*n* = 10). ****P < 0.0001 in (E) and (H), simple linear regression. ****P < 0.0001 in (F), survival curve comparison **P < 0.01.

To further prove the impact of NAC1 on autoimmunity, we next compared the response of the WT and NAC1^−/−^ mice to induction of autoimmune arthritis and colitis. Type II collagen was used to induce arthritis (*11*), and dextran sulfate sodium (DSS) was given to mice to induce colitis (*12*). We found that NAC1^−/−^ mice were significantly tolerant to induction of autoimmune arthritis and colitis (Fig. 4). In the collagen-induced arthritis model (CIA) (*11*), a significantly lower occurrence of CIA was observed in NAC1^−/−^ mice than in the littermate controls, as determined by the histologic evidence (Fig. 4A), disease incidence (Fig. 4B; *P* < 0.0001), and disease score (Fig. 4C; *P* < 0.0001). Tolerance to autoimmunity induction was recapitulated in a colitis model in which mice were given drinking water containing DSS. WT mice developed autoimmune colitis within 3 to 4 days following DSS administration and showed visible signs of illness including hunched back, raised fur, symptoms of sepsis, and reduced mobility because of diarrhea and anemia; notably, the occurrence of colitis declined remarkably in NAC1^−/−^ mice (Fig. 4, D to H). In NAC1^−/−^ mice, the body weight loss (Fig. 4E), survival (Fig. 4F), colon shrinkage (Fig. 4G), and the disease activity index (Fig. 4H) were all significantly improved as compared with WT animals (*P* < 0.0001). In both disease models, there were considerably larger amounts of proinflammatory immune cells in the joint (Fig. 4A) and the colon (Fig. 4D) tissues of WT mice than in those of NAC1^−/−^ animals (arrows), suggesting that weakened immune response may account for the insusceptibility of NAC1^−/−^ mice to induction of autoimmunity. We recognize that NAC1^−/−^ mice have NAC1 deficiency in all the immune cells such as macrophages, dendritic cells, B cells, and CD8^+^ T cells, which might also be involved in the pathogenesis of autoimmune arthritis and colitis. Nevertheless, as NAC1^−/−^ mice showed enhanced numbers and functions of T_regs_ (Figs. 1 and 3) and T_regs_ have a unique capacity to suppress immune response, the tolerance to the induction of autoimmune diseases observed in NAC1^−/−^ mice (Fig. 4) could be a consequence of the enhanced T_reg_ stability.

### DNA methylation of *FoxP3* in T_regs_ remains intact in the absence of NAC1

Next, we sought to understand how NAC1 regulates the development and function of T_regs_. As FoxP3 is a transcription factor essential for the establishment and maintenance of the T_reg_ phenotype and its activation is reported to be mediated by the promoter DNA demethylation (*13*), we asked whether the effect of NAC1 on T_regs_ could be due to the modulation of *FoxP3* promoter DNA methylation.

**Fig. 5.**
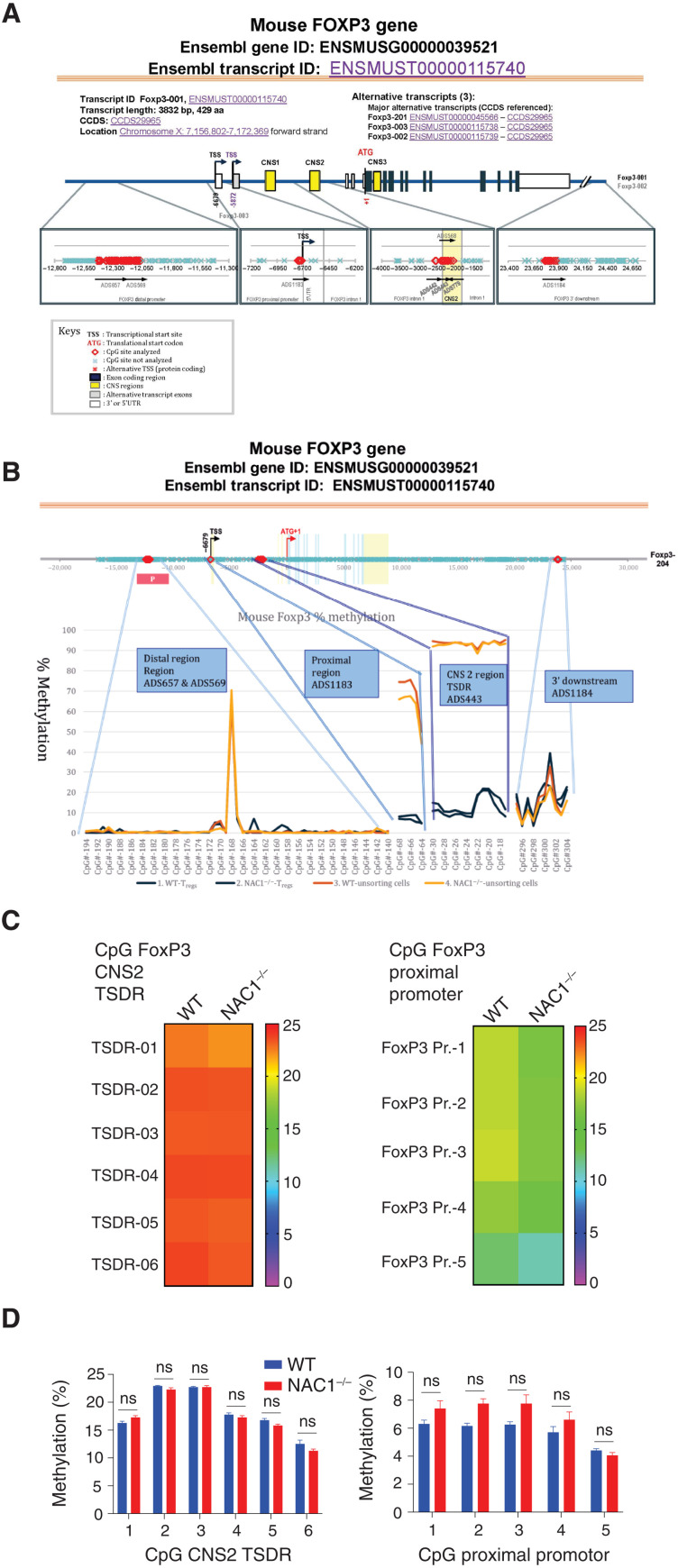
CpG motif analyses indicate that FoxP3 DNA methylation is not regulated by NAC1. Naive WT and NAC1^−/−^ CD4^+^CD25^+^ T_regs_ were purified and used in these analyses. (**A**) Diagram of mouse FoxP3 DNA methylation. Seven CpG sites of FoxP3 regulators (ADS657, ADS569, ADS1183, ADS442, ADS443, ADS779, and ADS1184) were analyzed. (**B**) Four CpG regions of FoxP3 regulators, including Distal Region (Regions ADS657 and ADS569), Proximal Region (ADS1183), CNS 2 Region (TSDR ADS443), and 3′ Downstream (ADS1184), were analyzed. (**C**) Genomic DNA of the lymphocytes from LNs and spleen of WT or NAC1^−/−^ mice was analyzed for methylation status of CNS2 (TSDR) and FoxP3 proximal promoter, respectively. The degree of methylation at each CpG motif is depicted according to the color code. A representative is shown. (**D**) Percentage of methylation of each FoxP3 CpG based on two experiments. *P* > 0.05; unpaired *t* test.

Primarily, activation of *FoxP3* is associated with selective demethylation of an evolutionarily conserved element within the *FoxP3* locus named TSDR (T_reg_-specific demethylated region) (*13*, *14*). To determine whether the effect of NAC1 on T_regs_ is mediated through promoter DNA methylation of *FoxP3* gene, we performed an analysis of TSDR demethylation. A panel of the *FoxP3* loci (ADS779, ADS657, ADS569, ADS442, ADS443, ADS1183, and ADS1184) between WT and NAC1^−/−^ T_regs_ were analyzed by the Targeted NextGen Bisulfite Sequencing (Fig. 5A), and the CpG DNA methylation of *FoxP3* in the four regions including Distal Region (ADS657 and ADS569), Proximal Region (ADS1183), CNS2 Region (ADS443), and 3′ Downstream Region (ADS1184) was determined (Fig. 5B). No substantial differences of the CpG DNA methylation in the four regions were found between WT and NAC1^−/−^ T_regs_ (Fig. 5C and table S1). The two bar graphs representing the specific methylation percentages of each *FoxP3* CpG in WT and NAC1^−/−^ T_regs_ are comparably presented from two experiments (Fig. 5D; *P* > 0.05). These results indicate that NAC1 does not have detectable effects on epigenetic silencing via DNA methylation in the TSDR region of *FoxP3*.

### *FoxP3* transcription in T_regs_ is unaltered in the absence of NAC1

**Fig. 6.**
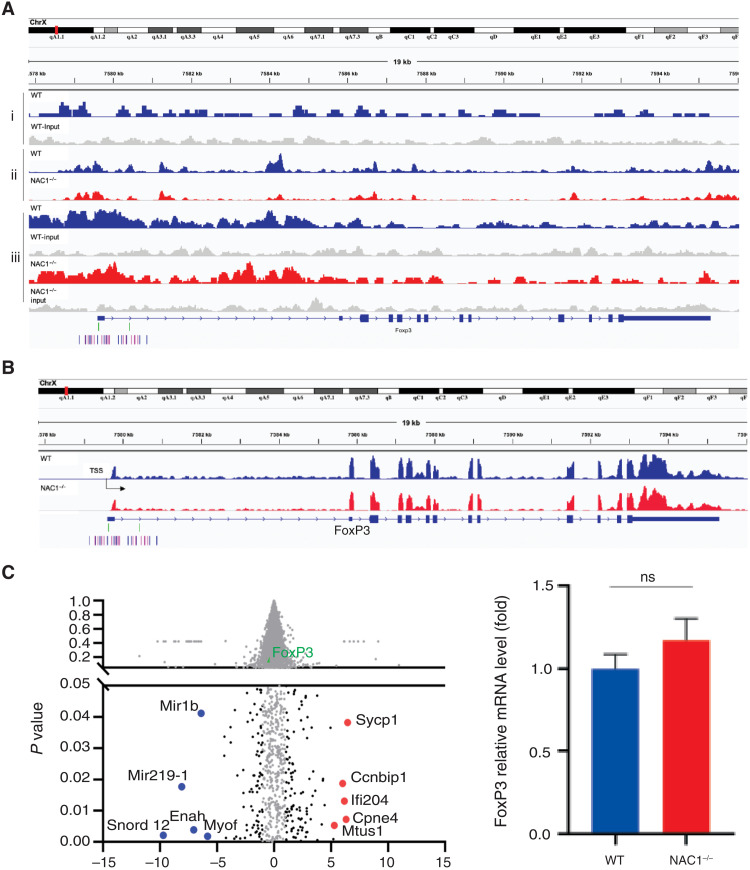
Combined analyses of ChIP-seq, ATAC-seq, and RNA sequencing indicate that FoxP3 transcription is not regulated by NAC1. (**Ai**) NAC1 ChIP-seq. Approximately 100 differential peaks (NAC1 versus input) were identified using HOMER. NAC1 peak density was not enriched within the regulatory elements of FoxP3. The genes targeted by NAC1-enriched islands in WT T_regs_ were presented. Representative genomic regions (promotor and CNS1-3) show NAC1 enrichment. Normalized ChIP-seq reads (bigWig) and enriched islands (bed) are shown. (**Aii**) ATAC-seq. Differential accessibility at the FoxP3 gene locus in WT T_regs_ compared with NAC1^−/−^ T_regs_ is presented. There were more than 10,000 differential ATAC-seq peaks in total, but there were not any significant differential accessibility at the FoxP3 gene locus in WT T_regs_ as compared with NAC1^−/−^ T_regs_. (**Aiii**) FoxP3 ChIP-seq. The genes targeted by FoxP3-enriched islands in WT or NAC1^−/−^ T_regs_ are presented. (**B**) RNA sequencing. FoxP3 was not differentially expressed in NAC1^−/−^ T_regs_ among ~200 differentially expressed genes, i.e., FoxP3 gene was identically expressed in WT T_regs_ and NAC1^−/−^ T_regs_. (**C**) Volcano plot (left) and summarized mRNA change between WT and NAC1^−/−^ T_regs_ (right). Black dots represent the significant changed genes (log_2_ fold change >2, *P* < 0.05). Red dots represent the top 5 up-regulated genes in NAC1^−/−^ T_regs_, and blue dots represent the top 5 down-regulated genes in NAC1^−/−^ T_regs_ compared to the WT. FoxP3 was marked as a green dot. All results shown are the representative of three identical experiments.

As NAC1 is a transcriptional coregulator, we wanted to know whether NAC1 affects the transcription of *FoxP3*. To compare *FoxP3* transcription in T_regs_ with or without NAC1, we first examined the possible enrichment and direct binding of NAC1 at the *FoxP3 *genetic locus in WT T_regs_, using chromatin immunoprecipitation coupled with deep sequencing (ChIP-seq). NAC1-associated chromatins were pulled down from the CD4^+^CD25^+^ T_regs_ of C57BL/6 mice, followed by a high-throughput sequencing. The results showed that the peak density of NAC1 was not enriched within regulatory elements of *FoxP3* (Fig. 6Ai and fig. S5). Assay for transposase accessible chromatin sequencing (ATAC-seq) identified a total of over 10,000 differential ATAC-seq peaks in WT and NAC1^−/−^ T_regs_, but no significantly differential accessibility was found at the *FoxP3 *gene locus (Fig. 6Aii). *FoxP3* ChIP-seq validated that *FoxP3* enrichment was not affected by NAC1 expression (Fig. 6Aiii). Furthermore, RNA sequencing (RNA-seq) showed that among ~200 differentially expressed genes, *FoxP3* was not differentially expressed in WT and NAC1^−/−^ T_regs_ (Fig. 6, B and C). These results suggest that the effect of NAC1 on FoxP3 expression is not mediated at the transcription level.

### NAC1 confines FoxP3 expression by promoting its deacetylation and destabilization

**Fig. 7.**
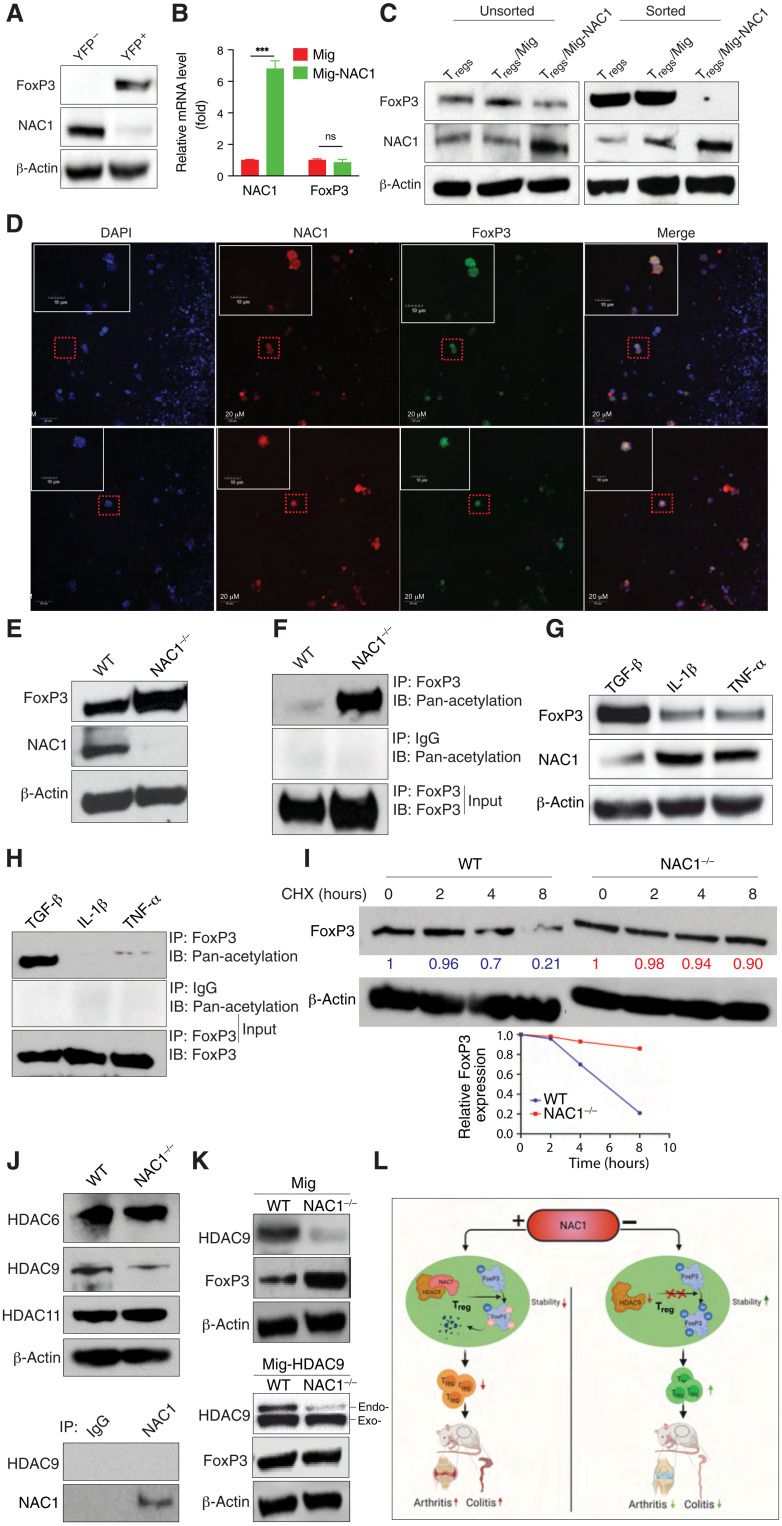
Up-regulation of NAC1 by proinflammatory cytokines breaks immune tolerance via down-regulation of FoxP3. (**A**) NAC1 and FoxP3 in T_regs_ from the FIR reporter mice by immunoblots. (**B**) NAC1 and FoxP3 mRNA of WT T_regs_ generated in vitro and with ectopic expression of NAC1 by reverse transcription polymerase chain reaction. ***P < 0.001. (**C**) FoxP3 protein of the sorted T_regs_ generated in vitro and with ectopic expression of NAC1 by immunoblots. (**D**) Immunofluorescence staining of 4′,6-diamidino-2-phenylindole, NAC1, and FoxP3 in T_regs_ generated in vitro. (**E**) Expressions of FoxP3 and NAC1 in T_regs_ from WT and NAC1^−/−^ mice by immunoblots. (**F**) FoxP3 was immunoprecipitated and examined for its acetylation of lysine. (**G**) WT T_regs_ were cultured in the presence of various cytokines for 12 hours, and then the expressions of FoxP3 and NAC1 were analyzed by immunoblots. (**H**) FoxP3 from WT T_regs_ treated in (G) was immunoprecipitated and examined for its acetylation. (**I**) WT or NAC1^−/−^ T_regs_ were treated with cycloheximide (150 μg/ml) and chased for the indicated hours. FoxP3 protein level was analyzed by immunoblotting. (**J**) HDAC expression. (**K**) HDAC9 and FoxP3 of the sorted WT or NAC1^−/−^ T_regs_ generated in vitro and with ectopic expression of either the control Mig vector or human HDAC9 vector were analyzed by immunoblots. All the data shown are the representative of three identical experiments. (**L**) Proposed model of regulation of FoxP3 by NAC1 in immunity.

Notably, our comparison of the FoxP3^+^ [yellow fluorescent protein–positive (YFP^+^)] T_regs_ with the FoxP3^−^ (YFP^−^) CD4^+^ T cells from FoxP3-IRES-mRFP (FIR) reporter mice revealed that the FoxP3^+^ T_regs_ expressed a low level of NAC1 but a high level of FoxP3; by contrast, the FoxP3^−^ CD4^+^ T cells had a high expression of NAC1 (Fig. 7A). Ectopic expression of NAC1 (Fig. 7B) in WT T_regs_ resulted in a reduction of FoxP3 protein (Fig. 7C). These results disclose an inverse relationship between the expression of NAC1 and FoxP3 in T_regs_. Moreover, colocalization of NAC1 and FoxP3 was observed in the nuclei of T_regs_ (Fig. 7D) and iT_regs_ (fig. S6). These results suggest that NAC1 confines FoxP3 expression.

Posttranslational modifications such as acetylation have a critical role in preventing proteasome-mediated degradation of FoxP3 and maintaining its functional activity (*15*, *16*), and in our previous study, we found that NAC1 can promote deacetylation of certain protein through its interaction with histone deacetylases (HDACs) (*7*). Thus, the acetylation of FoxP3 protein in NAC1^−/−^ T_regs_ and WT T_regs_ was examined. Figure 7 (E and F) shows that both FoxP3 and acetylated FoxP3 proteins were up-regulated in NAC1^−/−^ T_regs_ as compared with the WT T_regs_; in particular, acetylation of FoxP3 was robustly enhanced in NAC1^−/−^ T_regs_ than that of the WT T_regs_ (Fig. 7F). Conspicuously, in the T_regs_ treated with the proinflammatory cytokines IL-1β or tumor necrosis factor–α (TNF-α), a vigorous increase in NAC1 expression but evident decreases in both FoxP3 (Fig. 7G) and acetylated FoxP3 (Fig. 7H) were observed. Therefore, it is likely that stabilization of the acetylated FoxP3 protein accounts for the elevated amount of this key transcription factor in NAC1^−/−^ T_regs_, and in the T_regs_ treated with proinflammatory cytokines, up-regulation of NAC1 may induce deacetylation and destabilization of FoxP3. The pulse-chase experiments demonstrated that the turnover of FoxP3 protein was much faster in WT T_regs_ than in NAC1^–/–^ T_regs_ (Fig. 7I). HDACs have been reported to modulate the suppressive function of T_regs_ (*17*); therefore, we determined which HDAC may be involved in the NAC1-induced FoxP3 deacetylation. We examined the expressions of HDAC6, HDAC9, and HDAC11, the deacetylases known to affect the stability of FoxP3 and T_reg_ fitness (*18*). We demonstrated the compromised HDAC9 expression in NAC1^−/−^ T_regs_ as compared to WT T_regs_ but did not detect the direct interaction between NAC1 and HDAC9 (Fig. 7J). Moreover, ectopic expression of HDAC9 in NAC1^−/−^ T_regs_ resulted in a reduction of FoxP3 protein, which showed a similar level to that in WT T_regs_ (Fig. 7K). Our results suggest a role of HDAC9 in the regulation of the NAC1-mediated FoxP3 stability. Collectively, these results imply that NAC1 negatively regulates FoxP3 stability via its effect on deacetylation of this protein, thus weakening immune tolerance.

## DISCUSSION

Autoimmune diseases such as type 1 diabetes, rheumatoid arthritis, ulcerative colitis, and Crohn’s disease are presumed to result from interaction between genetic and environmental factors and to be a consequence of compromised immune tolerance versus adaptive immune response; however, how impaired balance between immune response and tolerance is triggered and the mechanisms by which tolerance is established and maintained remain elusive. How the stability of FoxP3 and suppressor T_regs_ are regulated is an important theme in T_reg_ biology. Using CRISPR screening, a recent study revealed several modulators of FoxP3 expression, and these modulators might be further explored as potential targets for immunotherapy (*19*). In this study, we identified NAC1 as a critical determinant of immune tolerance. We show that NAC1^−/−^ mice are substantially tolerant to the induction of autoimmunity, as evidenced by the significantly decreased occurrences of autoimmune arthritis and colitis (Fig. 4). We further show that the promoting effect of NAC1 on autoimmunity is mediated through its negative regulation of the stability of T_reg_ and FoxP3 (Fig. 7).

Although the promoter DNA methylation of *FoxP3* gene has been reported to be associated with the stability and function of T_regs_ (*13*, *20*–*22*), the effects of NAC1 on T_regs_ do not appear to be associated with alterations in DNA methylation of *FoxP3* gene. We compared the DNA methylation of *FoxP3* gene in WT and NAC1^−/−^ T_regs_ and examined the DNA methylation of *FoxP3* in the TSDR of CNS2 (ADS443) and *FoxP3* proximal promoter region (ADS1183; Fig. 5). As a control, *FoxP3* DNA methylation was similar in the unsorted lymphocytes from the LNs and spleen of NAC1^−/−^ mice (table S1). In these experiments, greater than 100 CpG sites in the *FoxP3* DNA promoter regions showed that there was no significant difference between WT and NAC1^−/−^ T_regs_, indicating that NAC1 does not affect the DNA methylation of *FoxP3* gene. Moreover, we found that NAC1 does not act as a transcriptional regulator (Fig. 6). Instead, we show that NAC1 can interfere with the acetylation resulting in degradation of FoxP3 protein (Fig. 7). Thus, the role of NAC1 in the posttranscriptional regulation of FoxP3 may account for the up-regulation of FoxP3 in NAC1^−/−^ T_regs_.

T_reg_ stability is vital to the maintenance of immune tolerance but is often altered in autoimmunity; however, how destabilization of T_regs_ occurs in autoimmune diseases remains elusive. In autoimmune arthritis, TNF-α plays a more important role in triggering events leading to inflammation both locally and systemically, whereas IL-1 is more involved at the local level in the processes leading to cartilage and bone destruction and in impeding cartilage repair. Nevertheless, IL-1 and TNF-α strongly synergize in numerous biological functions, and simultaneous blockade of IL-1 and TNF-α provides favorable effects in suppressing arthritis development, suggesting the importance of both cytokines (*23*, *24*). Our results reported here, which show the concomitant up-regulation of NAC1 and down-regulation of FoxP3 in T_regs_ treated with the proinflammatory cytokines such as IL-1β and TNF-α (Fig. 7, G and H), provide at least a partial explanation for this question. On the basis of these findings, we speculate that the “basal” level of NAC1 in T_regs_ plays an important role in leashing the immune tolerance to keep the immune system vigilant to pathogens; inflammatory stimulation induces up-regulation of NAC1, and this, in turn, destabilizes FoxP3 and converts FoxP3^+^ T_regs_ to FoxP3^−^ T_regs_ that then become T helper 1 (T_H_1) or T_H_17 CD4^+^ T_effs_, further breaking tolerance and instigating strong immune response (Fig. 7L).

In this study, the role and importance of the NAC1-mediated regulation of FoxP3 in T_regs_ were largely determined in mice with complete knockout of *NAC1* gene. We demonstrated that NAC1 can modulate the stability of the FoxP3 protein, and loss of NAC1 enhances the functional activity of T_regs_. It shall be interesting to further investigate the functions of NAC1 in hematopoietic cells. Our data showing that NAC1 deletion is associated with reduced effector cell activity (figs. S2 and S3) open a question of whether impairment of the effector responses is also involved in the reduced inflammation caused by NAC1 deletion. The roles of NAC1 in other immune cells such as CD8^+^ T and CD4^+^ T_H_1 or T_H_17 effector cells warrant further investigation.

Because the mice that we used in this study were subjected to complete knockout of the *NAC1* gene, the deficiency of NAC1 in other cell types may also affect immune tolerance. For example, the role of NAC1 in other types of T cells such as CD8^+^ T cells, conventional CD4^+^ T cells (i.e., T_H_1, T_H_2, and T_H_17), and innate immune cells including macrophages and dendritic cells remains unclear. To exclude the possibility that NAC1 deficiency in other types of cells may affect T_reg_ development, we performed the bone marrow chimera experiment in which the bone marrow cells (CD4^−^CD8^−^) from WT and NAC1^−/−^ mice were transferred into x-ray–irradiated WT mice, and 6 weeks later, we euthanized the mice and isolated the spleen, LNs, and thymus for examining T_reg_ development. These experiments showed the comparable numbers of T_regs_ in the thymus of the chimera receiving either WT or NAC1^−/−^ bone marrow cells. However, we observed that the bone marrow transplants from NAC1^−/−^ mice generated greater numbers of T_regs_ in the LNs and spleen than those from WT (fig. S7), which is like that we classified in NAC1^−/−^ mice. This result also supports the conclusion that in the peripheral system, NAC1^−/−^ T_regs_ are more stable than WT cells.

As the stability of T_regs_ is vital to the maintenance of immune tolerance, the role of NAC1 in destabilization of suppressor T_regs_ provides a promising opportunity for therapeutic manipulation of their stability and function. We believe that therapeutic targeting of NAC1 to revitalize suppressor T_regs_ may be further exploited as a potentially novel tolerogenic strategy to treat autoimmune diseases. We have recently identified and characterized a small-molecule inhibitor of NAC1, NIC3, through a high-throughput screening and showed that this compound can effectively promote the proteasome-mediated degradation of NAC1 protein (*25*). We anticipate that inhibiting NAC1 by pharmacologic approaches shall provide further insights into the feasibility and effectiveness of NAC1-based modulation of T_reg_ stability for therapeutic purposes.

## METHODS

### Cell lines and mice

C57BL/6 (B6), *Rag1^−/−^*, and FIR reporter mice were purchased from the Jackson Laboratory (Bar Harbor, ME). NAC1^−/−^ mice were generated by J. Wang and crossed in the C57BL/6 background for more 10 generations (*6*, *26*). All the animal experiments were performed in compliance with the regulations of The Texas A&M University Animal Care Committee (IACUC no. 2018-0065) and in accordance with the guidelines of the Association for the Assessment and Accreditation of Laboratory Animal Care.

### T cell culture

T cells were cultured in 48-well plates containing 1 ml of RPMI 1640 (Invitrogen) with 10% fetal calf serum (Omega Scientific, CA). T cell isolation kits including mouse CD4^+^ (no. 130-104-454), CD8a^+^ (no. 130-104-075) and CD4^+^ CD25^+^ T_reg_ (no. 130-091-041), T cell activation/expansion kit (no. 130-093-627), and T_reg_ expansion kit (no. 130-095-925) were purchased from Miltenyi Biotec (Auburn, CA). Recombinant mouse TGF-β (no. 763104), IL-1β (no. 575106), and TNF-α (no. 575206) were obtained from BioLegend (San Diego, CA).

### Cytokine secretion, cell recovery, and proliferation/cell division

IL-2 and interferon-γ were measured using ELISA after 48 hours of culture (*27*). Latent TGF-β1 (no. 433007, BioLegend) and IL-10 (no. 431411, BioLegend) were determined using ELISA after 48 hours of stimulation. In vitro T cell survival was determined using trypan blue exclusion. Proliferation/division of T cells was measured using the CellTrace CFSE Cell Proliferation Kit (no. C34554, Invitrogen).

### Metabolic assays

Purified CD4^+^ T_regs_ were plated in the Cell-Tak–coated Seahorse Bioanalyzer XFe96 culture plates (300,000 or 100,000 cells per well, respectively) in assay medium consisting of minimal, unbuffered Dulbecco’s modified Eagle’s medium supplemented with 1% bovine serum albumin (BSA) and 25 mM glucose, 2 mM glutamine (and 1 mM sodium pyruvate for some experiments). Basal rates were taken for 30 min, and then streptavidin-complexed anti-CD3bio at 3 mg/ml ± anti-CD28 at 2 mg/ml or PMA (phorbol 12-myristate 1-acetate; CAS 16561-29-8; Fisher) was injected and readings were taken for 1 to 6 hours. In some experiments, oligomycin (2 mM), carbonyl cyanide p-trifluoromethoxyphenylhydrazone (0.5 mM), 2-deoxy-d-glucose (10 mM), and rotenone/antimycin A (0.5 mM) were injected to obtain maximal respiratory and control values. Because OCR or ECAR values tend to vary among experiments, both a representative trace and normalized data (calculated as the difference between maximal and basal OCR or ECAR values) were shown in the figures.

### In vitro mouse T_reg_ generation

Naive CD4^+^CD25^−^ T cells from the LNs and spleen of WT or NAC1^−/−^ mice were incubated with the indicated reagents including TGF-β in the CellXVivo Mouse T_reg_ Cell Differentiation Kit (no. CDK007, R&D Systems) for 5 days.

### In vitro T_reg_ suppression assay

CD4^+^ CD25^+^ T_regs_ were cocultured with the CFSE-labeling CD4^+^ CD25^−^ responder T cells from the pooled LNs and spleen of C57BL/6 mice in various ratios. To stimulate T cells, the mixed T cells were treated with the T Cell Activation/Expansion Kit (no. 130-093-627; Miltenyi Biotec). As controls, CD4^+^ CD25^+^ T_regs_ and CD4^+^ CD25^−^ responder T cells were cultured without any stimulus. Suppression of responder T cells was determined by measuring CFSE dilution.

### T cell transfer model of colitis

Naive CD4^+^ T_effs_ (CD45RB^hi^CD25^−^) from B6 mice and CD4^+^ T_regs_ (CD45RB^lo^CD25^+^) from WT or NAC1^−/−^ mice were purified using a high-speed cell sorter. Naive CD4^+^ T_effs_ (6 × 10^5^ cells per mouse) without or with T_regs_ (2 × 10^5^ cells per mouse) were then intraperitoneally transferred into *Rag*1^−/−^ mice. Body weights were recorded twice a week. When loss of body weight exceeded 20% after transfer, the host mice were euthanized.

### Retroviral transduction

Full-length cDNA of NAC1 provided by I.-M. Shih and T.-L. Wang (John Hopkins University (*28*) or human HDAC9 was subcloned into the Mig vector containing green fluorescent protein for retroviral transduction of mouse T_regs_ (*29*).

### Antibodies and reagents

Phycoerythrin (PE)-, PE/Cy7, Alexa 647, allophycocyanin (APC)-, or APC/Cy7-conjugated anti-mouse CD4 (GK1.5), CD8 (53-6.7), CD25 (3C7), CD45RB (C363-16A), CD25 (3C7), CD44 (IM7), CD117 (2B8), TCRVβ (H57-597), TGF-β1 (TW7-16B4), FoxP3 (MF-14), and acetylated lysine (15G10; no. 623402) were purchased from BioLegend (San Diego, CA). Rabbit NAC1 (no. 4183), HDAC6 (no. 7612), and β-actin (no. 8457) antibodies were purchased from Cell Signaling (Beverly, MA). Rabbit anti-NAC1 antibody (ab29047) for immunoprecipitation was obtained from Abcam (Cambridge, MA). Mouse HDAC9 (no. sc-398003) and HDAC11 (no. sc-390737) antibodies were purchased from Santa Cruz Biotechnologies (Dallas, TX). Cycloheximide were purchased from Sigma-Aldrich Corporation (Sigma-Aldrich, St. Louis, MI).

### Immunoprecipitation and immunoblotting

Cells were lysed in ice-cold radioimmunoprecipitation lysis buffer (no. 89900, Thermo Fisher Scientific, MA) for 30 min. Insoluble materials were removed, and the lysates were used for Western blotting or immunoprecipitated overnight with an antibody such as anti-FoxP3 antibody followed by incubation with protein G agarose beads at 4°C for 2 hours. The washed immunoprecipitates were boiled in SDS sample buffer, and the protein content was determined by the Bio-Rad protein assay kit (no. 5000002, Bio-Rad, Hercules, CA). Equal amounts (30 to 50 μg) were loaded onto 4 to 12% NuPage bis-tris precasting gels (SDS–polyacrylamide gel electrophoresis), transferred onto polyvinylidene difluoride membrane (Invitrogen), and immunoblotted. All blots were developed with the ECL immunodetection system (no. 426319, BioLegend, CA).

### Reverse transcription polymerase chain reaction

Retrovirally transduced T_regs_ with Mig or Mig-NAC1 were unsorted or sorted, and total RNA was extracted from the T_regs_ using QIAgen RNeasy mini kits. Samples were subjected to reverse transcription using a high-capacity cDNA synthesis kit (Applied Biosystems). Polymerase chain reaction (PCR) analysis was performed using TaqMan real-time PCR (Thermo Fisher Scientific). Primers used were as follows: FoxP3, 5′-CCCAGGAAAGACAGCAACCTT-3′ (forward) and 5′-TTCTCACAACCAGGCCACTTG-3′ (reverse); NAC1, 5′-TGC TTA GTT AAC TTA CTG CAG GGC TTC AGC CGA-3′ (forward) and 5′-TAA GCA CTC GAG ATG GCC CAG ACA CTG CAG ATG-3′ (reverse).

### CpG DNA methylation

CpG DNA methylation was analyzed by bisulphite treatment of ribonuclease (RNase)–treated genomic DNA, followed by PCR amplification and pyrosequencing (Pyro Q-CpG), which was performed by EpigenDX. Eight mouse genes, including FoxP3, Ctla4, Ikzf2, Ikzf4, Tnfrsf18, Il2ra, Cd274, and Irf4, were screened for methylation percentage in various regulatory regions. Sequence analyses for FoxP3 were as follows: *FoxP3* promoter, *FoxP3* CNS2, and *FoxP3* 3′ region.

### RNA-seq

T_regs_ were mechanically disrupted and homogenized using a Mini-BeadBeater-8 (BioSpec Products, Bartlesville, OK). RNA was extracted using an RNeasy Mini kit (Qiagen, Valencia, CA). RNA concentration and integrity were measured using an Agilent 2100 Bioanalyzer (Agilent Technologies, Santa Clara, CA). All samples had an RNA Integrity Value (RIN) of >7.5. RNA-seq libraries were prepared using the Illumina TruSeq Stranded mRNA Library Prep Kit (Illumina, San Diego, CA) and sequenced on an Illumina HiSeq 2500 Sequencer (Illumina, San Diego, CA) as 75–base pair (bp) paired-end reads.

### CHIP-seq

ChIP was performed as described (*30*), with some modifications. T_regs_ were subjected to sonication using a Bioruptor Pico sonication device (Diagenode) to obtain 100- to 500-bp chromatin fragments. A total of 250 μg of sonicated chromatin fragments was incubated with 10 μg of NAC1 antibody for cross-linking with magnetic beads (no. 11201D, Dynabeads M280 sheep anti-mouse immunoglobulin G, Dynal Biotech, Invitrogen). The cross-linked samples were reversed at 65°C overnight, and the precipitated DNA was treated with RNase A and proteinase K and then purified using the QIAquick PCR Purification Kit (Qiagen). The DNA libraries were prepared following the guidelines from Illumina (Fasteris Life Sciences; Plan-les-Ouates, Switzerland). Input DNA was sequenced and used as a control. The DNA libraries were sequenced on Illumina HiSeq2500, producing 25 to 35 million reads per sample.

### ATAC-seq

T_regs_ were freshly dissected and processed for ATAC-seq. In brief, the tissues were resuspended in 1 ml of lysis buffer (1× phosphate-buffered saline, 0.2% NP-40, 5% BSA, 1 mM dithiothreitol, and protease inhibitors), followed by Dounce homogenization with a loose pestle using 20 strokes. The lysates were then filtered through a 40-μm cell strainer, and the nuclei were collected by centrifugation at 500*g* for 5 min. Tagmentation was performed immediately according to the reported ATAC-seq protocol (*31*).

### Pulse-chase analysis

Isolated T_regs_ from WT or NAC1^−/−^ mice were activated and expanded with kits (no. 130-104-454 and no. 130-095-925; Miltenyi Biotec) and then treated with cycloheximide (150 μg/ml) for various periods of time. FoxP3 protein was analyzed by immunoblotting.

### Collagen-induced arthritis

C57BL/6 mice (4 months old) were injected at the base of the tail with 0.1 ml of emulsion containing 100 μg of bovine type II collagen (CII) (Chondrex, Redmond, WA, USA) in complete Freund’s adjuvant (Chondrex) using a 1-ml glass tuberculin syringe with a 26-gauge needle. Mice were assessed for arthritis in the paws (*32*).

### DSS-induced colitis

Colitis was induced in mice by oral ingestion of 3% DSS (SKU 02160110-CF; MP Biomedicals) in drinking water for 5 days. The severity of colitis activity was graded on designated dates as described (*33*). Body weight, occult or gross rectal bleeding, and feces consistency (on scales of 0 to 4) were monitored for each mouse. The resultant inflammatory bowel disease (IBD) disease activity index is the average of the scores of the colitis symptoms. The occult blood in mouse fecal samples was detected using Hemoccult Test Kit (Beckman Coulter Inc., Fullerton, CA).

### LPS-induced colitis

To induce colitis, B6.Thy1.1 Tg mice were intraperitoneally injected with LPS (20 μg/kg). In the following day, 3 × 10^6^ WT or NAC1^−/−^ T_regs_ were intravenously transferred into LPS-challenged mice via the tail vein. Seven days later, mice were euthanized, and the colons and spleens of the mice were dissociated. The length of the colon was measured, and the colons were processed for hematoxylin and eosin (H&E) staining and flow cytometric analysis.

### Histology and immunohistochemistry

Joint or gut tissues were fixed with 10% neutral formalin solution (VWR, West Chester, PA), and the fixed samples were prepared and stained with H&E as described (*34*). For immunofluorescence microscopy, the tissues were frozen in cryovials on dry ice immediately following resection. Cryo-sectioning and immunofluorescence staining were performed as described (*34*).

### Statistical analysis

Multiple unpaired *t* test, simple linear regression, and survival curve comparison were performed to analyze the differences between the groups, using GraphPad Prism (GraphPad Software, San Diego, CA); significance was set at 5%.

## Supplementary Materials

### This PDF file includes:

Extended Methods Generation of bone marrow chimera Figs. S1 to S7 Table S1

## Notes

View/request a protocol for this paper from *Bio-protocol*.
