# Supplementary material for: NAC1 modulates autoimmunity by suppressing regulatory T cell–mediated tolerance
Source: Sci Adv. 2022 Jun 29;8(26):eabo0183. doi: 10.1126/sciadv.abo0183 (PMC9242588; doi:10.1126/sciadv.abo0183)
Supplement: Supplementary file 1 — Extended Methods Generation of bone marrow chimera Figs. S1 to S7 Table S1 [file sciadv.abo0183_sm.pdf]

Science Advances
AAAS

Supplementary Materials for

NAC1 modulates autoimmunity by suppressing regulatory T cell–mediated tolerance

Jin-Ming Yang *et al.*

Corresponding author: Jin-Ming Yang, jyang@uky.edu; Jianlong Wang, jw3925@cumc.columbia.edu; Jianxun Song, jus35@tamu.edu

Sci. Adv. **8**, eabo0183 (2022)
DOI: 10.1126/sciadv.abo0183

This PDF file includes:

Extended Methods
Generation of bone marrow chimera
Figs. S1 to S7
Table S1

Extended Methods

Reagents

H-2K^b VACV B8R (TSYKFESV) Tetramer (#TB-M538-1, MBL), anti-mouse CD36 antibody (clone HM36, BioLegend), anti-human/mouse/rat NAC1 antibody (clone SWN-3, BioLegend), anti-human FoxP3 antibody (clone 206D, BioLegend), and L-(+)-Lactic acid (#ICN19022805, MP Biomedicals).

Murine Melanoma Model

WT or NAC1$^{-/-}$ Tregs (1 x 10^5) were injected *s.c.* in the flank region of the recipient mice inoculated with 1 x 10^6 B16 tumor cells. Tumor sizes were measured by a caliper and tumor volumes were calculated as: V=long diameter × short diameter^2 × 0.52 (*37*).

Generation of bone marrow chimera

To prepare B6. Thy1.1$^+$ Tg recipient mice for irradiation, drinking water was removed 12 hrs prior to irradiation. The mice were then X-ray irradiated (1000 rad) with a RS200 X-ray irradiator (Rad Source Technologies Inc. GA, USA). The bone marrow cells were harvested from femur of C57BL/6 Thy1.2$^+$ WT and NAC1$^{-/-}$ mice, using standard procedures. Red blood cells (RBCs) were lysed, and CD4$^+$ cells and CD8$^+$ cells were depleted by the negative selection using biotin conjugated anti-CD4 and anti-CD8 antibodies and streptavidin nanobeads. CD4$^+$ and CD8$^+$-depletions were analyzed by flow cytometry. Ten millions of CD4/CD8-depleted WT and NAC1$^{-/-}$ bone marrow cells were *i.v.* injected into the irradiated B6.Thy1.1$^+$ recipient mice. Sulfatrim (sulfamethoxazole and trimethoprim oral suspension)-containing drinking water (5ml/200ml) was

given to the irradiated animals for 2 weeks. After 6 weeks, the spleen, LNs and thymus were isolated, and cells were analyzed by flow cytometry to evaluate the development of Tregs.

Legends to Extended Data

Figure S1. NAC1$^{-/-}$ mice have a decreased percentage of TCRVβ cells in DN4 stage. The thymocytes from WT or NAC1$^{-/-}$ mice were analyzed by flow cytometry and calculated for numbers or percentages. (**A**) CD117 and TCR Vβ. The DN populations were analyzed for CD117 and TCRVβ. Data shown are the representative of three identical experiments. (**B**) TCRVβ and CD25. The DN4 populations were analyzed for TCR Vβ and CD25. Data are the representative of three identical experiments (N = 5). *, $p<0.05$, **, $p<0.01$, ***, $p<0.001$, Student's unpaired *t*-test.

Figure S2. NAC1$^{-/-}$ CD8^{+} T cells are defective in cytokine production and survival. (**A-B**) Percentages (**A**) and numbers (**B**) of CD8^{+} T cells from the pooled LNs and spleen of WT or NAC1$^{-/-}$ mice. Data shown are the representative of three identical experiments. The values represent mean ± S.D. (N = 3). *, $p<0.05$, Student's unpaired *t*-test. (**C-D**) Purified CD8^{+} T cells from the pooled LNs and spleen of WT or NAC1$^{-/-}$ mice were stimulated with anti-CD3 plus CD28 antibodies. (**C**) Cytokine production. ** $p<0.01$, Student's unpaired *t*-test. The values represent the mean ± S.D. (N = 3). (**D**) Cell recovery on various days. The numbers of T cells present on day 0 were assigned a value of 100%, and numbers surviving on various days were used to calculate the percentage recovery relative to day 0. Data shown are the mean ± S.E.M. of percentage change of a representative of three identical experiments (N = 3). **, $p<0.01$, Nested *t*-test).

Figure S3. Both NAC1$^{-/-}$ iTregs and naturally occurring Tregs (nTregs) produce more suppressive cytokines than WT iTregs and nTregs. WT or NAC1$^{-/-}$ iTregs and nTregs were examined productions of suppressive cytokines (IL-10 and TGF-β) by intracellular staining and flow cytometric analysis. (**A**) Productions of IL-10 and TGF-β. (**B**) MFI of IL-10 and TGF-β. Data

shown are the representative of three identical experiments. * $P<0.05$, ***, $P<0.0001$, ***, $P<0.00001$, Student's unpaired *t*-test.

Figure S4. NAC1$^{-/-}$ Tregs show enhanced suppressive function. WT or NAC1$^{-/-}$ Tregs (1 x 10^5) were injected *s.c.*in the flank region of the recipient mice with 1 x 10^6 B16 tumor cells on various days. (**A**) Tumor growth. Data shown are the mean ± S.E.M. of tumor sizes of a representative of three identical experiments (N = 6). ***, $P<0.0001$, simple linear regression. (**B**) Survival curves. Data shown are the representative of three identical experiments (N = 6). ns, $P>0.05$, Log-rank (Mantel-Cox) test.

Figure S5. FoxP3 elements regulated by NAC1 are located between Exon 2 and Exon 8. ChIP-seq analysis of naive CD4$^+$CD25$^+$ Tregs from the pooled LNs and spleen of WT mice. NAC1-enriched islands are shown. Representative genomic regions (Exon 2 and Exon 8) show NAC1 enrichment. Normalized ChIP-seq reads (bigWig) and enriched islands (bed) are shown. Results shown are the representative of three identical experiments

Figure S6. Co-localization of NAC1 and FoxP3 in the nuclei of Tregs. Immunofluorescent staining of DAPI, NAC1 and FoxP3 in Tregs generated *in vitro*. Arrows indicate overlays with DAPI, NAC1 and FoxP3. Data shown are the representatives of three identical experiments.

Figure S7. Thymic development of NAC1$^{-/-}$ Tregs in the bone marrow chimeras. Bone marrow cells (CD4$^-$CD8$^-$; Thy1.2$^+$) from WT and NAC1$^{-/-}$ mice were transferred into X-ray irradiated mice (Thy1.1$^+$). Six weeks later, the mice were euthanized, and the spleen, LNs and thymus were

isolated to examine Treg development using flow cytometry. (**A**) $CD4^+FoxP3^+$ cells. Data shown are the representatives of two identical experiments (N = 10). (**B**) $Thy1.1^+FoxP3^+$ cells. Data shown are the representatives of two identical experiments (N = 10). (**C**) Total numbers of cells. Data shown are the mean ± S.E.M. of a representative of two identical experiments (N = 10). ***, $p<0.001$; ns, no statistical difference, Student's unpaired *t*-test).

Table S1. Detailed CpG analysis of mouse FoxP3 DNA methylation. Seven CpG sites of FoxP3 regulators were analyzed.

Figure S1

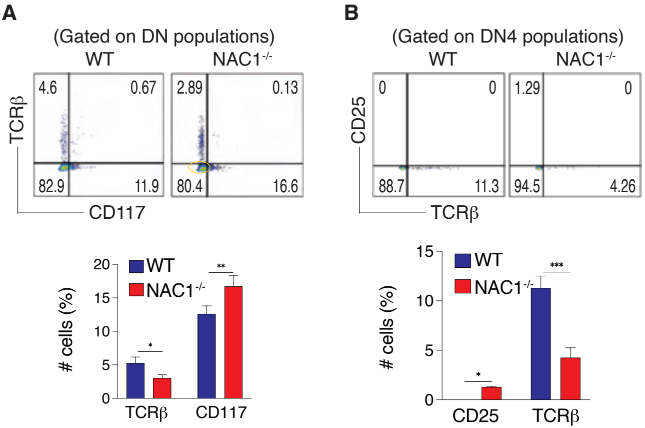
A
(Gated on DN populations)
WT
NAC1-/-
4.6
0.67
82.9
11.9
2.89
0.13
80.4
16.6
TCRβ
CD117
cells (%)
20
15
10
5
0
WT
NAC1-/-
*
**
TCRβ
CD117
B
(Gated on DN4 populations)
WT
NAC1-/-
0
0
88.7
11.3
1.29
0
94.5
4.26
CD25
TCRβ
cells (%)
15
10
5
0
WT
NAC1-/-
*
***
CD25
TCRβ

Figure S2

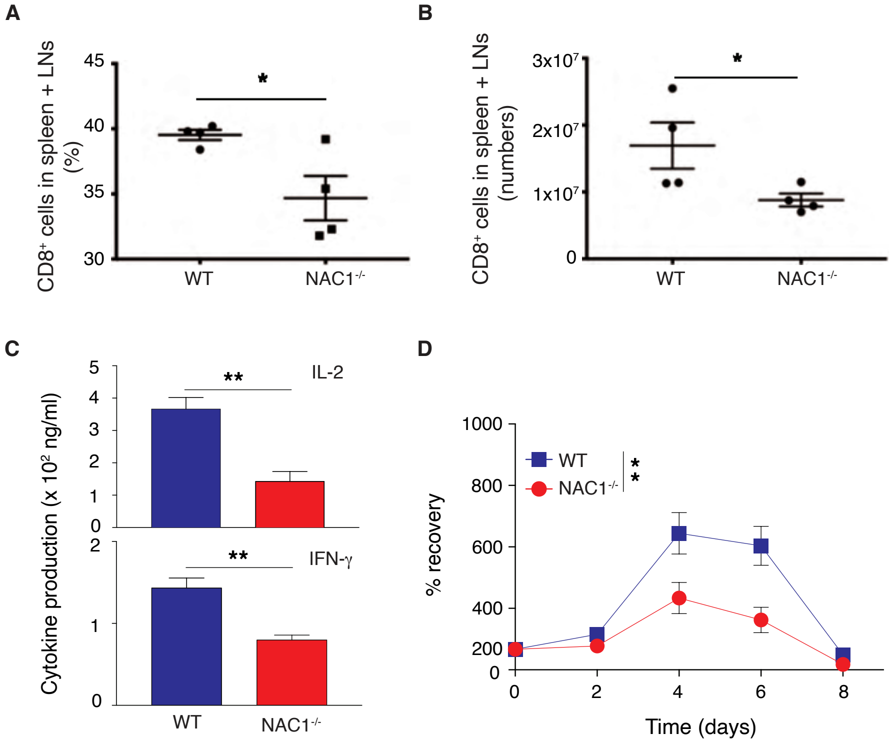

A
CD8+ cells in spleen + LNs (%)
45
40
35
30
*
WT
NAC1-/-
B
CD8+ cells in spleen + LNs (numbers)
3x10^7
2x10^7
1x10^7
0
*
WT
NAC1-/-
C
Cytokine production (x 10^2 ng/ml)
5
4
3
2
1
0
**
IL-2
2
1
0
**
IFN-γ
WT
NAC1-/-
D
% recovery
1000
800
600
400
200
0
WT
NAC1-/-
**
0
2
4
6
8
Time (days)

Figure S3

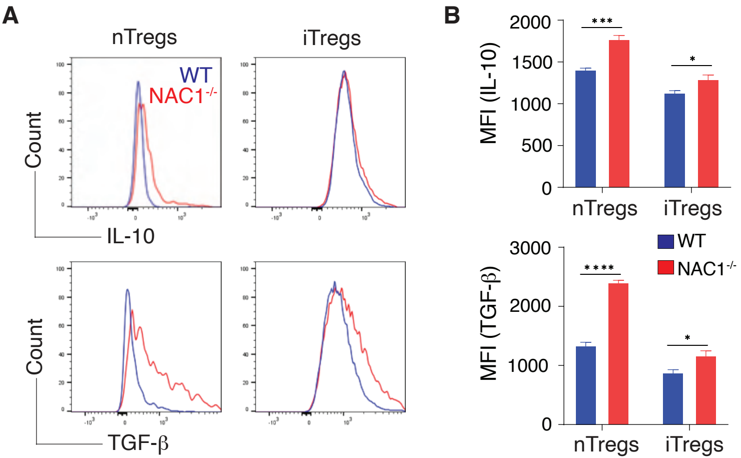

A
nTregs
iTregs
WT
NAC1-/-
Count
IL-10
Count
TGF-β
B
MFI (IL-10)
2000
1500
1000
500
0
***
*
nTregs
iTregs
MFI (TGF-β)
3000
2000
1000
0
****
*
WT
NAC1-/-
nTregs
iTregs

Figure S4

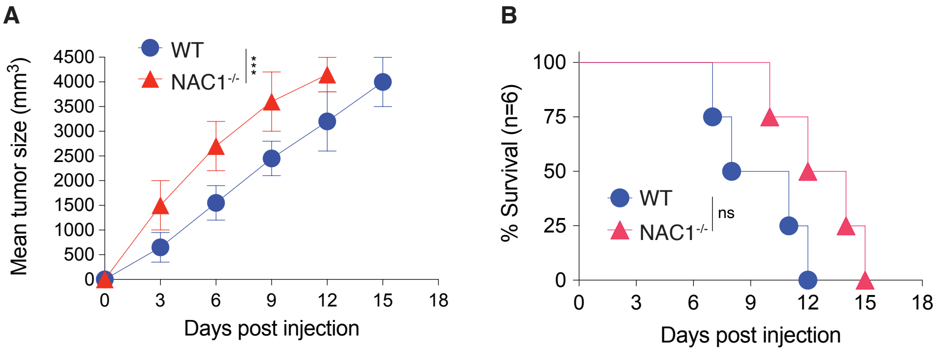
A
WT
NAC1-/-
***
Mean tumor size (mm3)
4500
4000
3500
3000
2500
2000
1500
1000
500
0
0
3
6
9
12
15
18
Days post injection
B
% Survival (n=6)
100
75
50
25
0
WT
NAC1-/-
ns
0
3
6
9
12
15
18
Days post injection

Figure S5

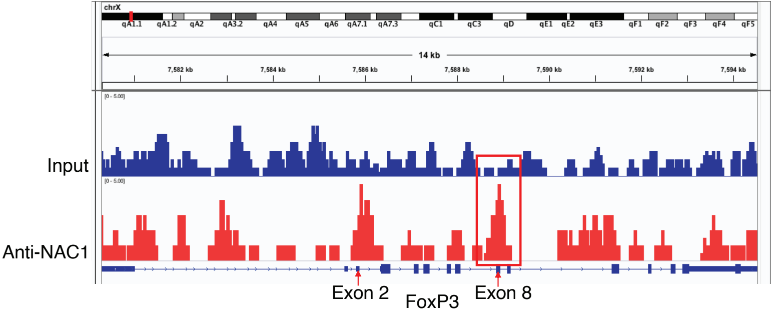

chrX
qA1.1
qA1.2
qA2
qA3.2
qA4
qA5
qA6
qA7.1
qA7.3
qC1
qC3
qD
qE1
qE2
qE3
qF1
qF2
qF3
qF4
qF5
14 kb
7,582 kb
7,584 kb
7,586 kb
7,588 kb
7,590 kb
7,592 kb
7,594 kb
[0 - 5.00]
Input
[0 - 5.00]
Anti-NAC1
Exon 2
FoxP3
Exon 8

Figure S6

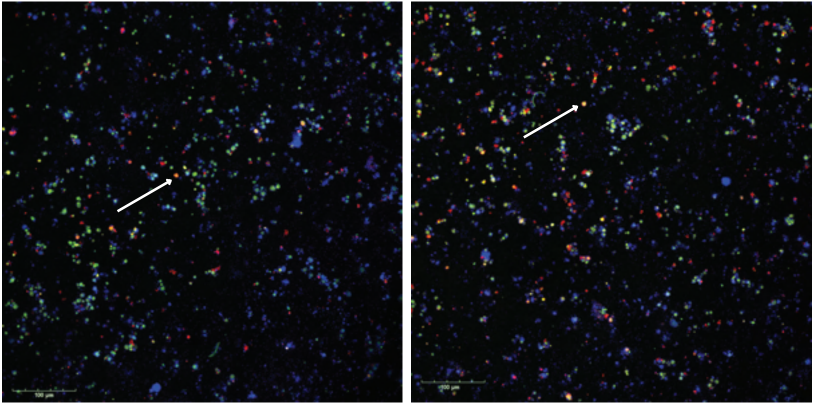

DAPI/NAC1/FoxP3

Figure S7

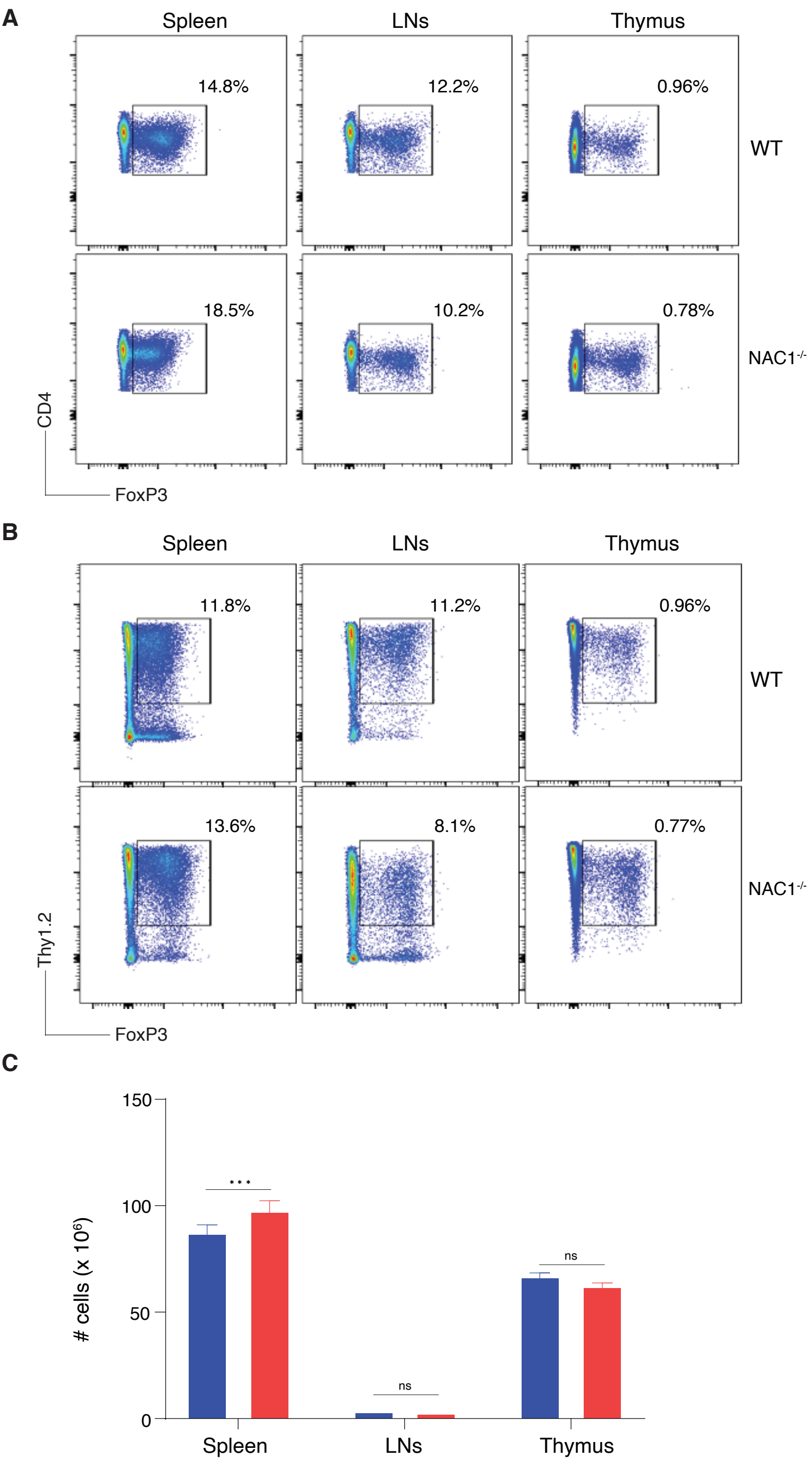
A
Spleen
LNs
Thymus
14.8%
12.2%
0.96%
WT
18.5%
10.2%
0.78%
NAC1-/-
CD4
FoxP3
B
Spleen
LNs
Thymus
11.8%
11.2%
0.96%
WT
13.6%
8.1%
0.77%
NAC1-/-
Thy1.2
FoxP3
C
150
100
50
0
cells (x 10^6)
***
ns
ns
Spleen
LNs
Thymus

Table S1 Detailed CpG analysis of mouse FoxP3 DNA methylation

	Mouse FoxP3 DNA Methylation					
Gene	Assay ID	Assay Location	From ATG	From TSS	Coordinates	# of CpG
Foxp3	ADS657	5-Upstream	-12434 to -12224	-5755 to -5545	ChrX:7573921 - 7574131	25
Foxp3	ADS1183	5-Upstream	-6750 to -6714	-71 to -35	ChrX: 7579605 - 7579641	5
Foxp3	ADS569	5-Upstream	-12210 to -12059	-5531 to -5380	ChrX: 7574145 - 7574296	30
Foxp3	ADS443	Intron 1	-2405 to -2353	4275 to 4327	ChrX:7583950 - 7584002	3
Foxp3	ADS442	Intron 1	-2555 to -2533	4125 to 4147	ChrX: 7583800 - 7583822	2
Foxp3	ADS779	Intron 1	-2145 to -2075	4535 to 4605	ChrX: 7584210 - 7584280	2
Foxp3	ADS1184	3-Downstream	23773 to 23905	30452 to 30584	ChrX: 7610127 - 7610259	10

Assay ID	Assay Location	From ATG	From TSS	NCBI37/mm9	GRCm38/mm10	# of CpG
ADS657-FS1	Distal Promoter	-12434 to -12401	-5755 to -5722	ChrX:7151047-7151080	ChrX:7573921-7573954	6
ADS657-FS2	Distal Promoter	-12359 to -12353	-5680 to -5674	ChrX:7151122-7151128	ChrX:7573996-7574002	2
ADS657-FS3	Distal Promoter	-12335 to -12253	-5656 to -5574	ChrX:7151146-7151228	ChrX:7574020-7574102	15
ADS657-FS4	Distal Promoter	-12231 to -12225	-5551 to -5545	ChrX:7151251-7151257	ChrX:7574125-7574131	2
ADS569-FS1	Distal Promoter	-12210 to -12136	-5531 to -5457	ChrX:7151271-7151345	ChrX:7574145-7574219	15
ADS569-FS2	Distal Promoter	-12118 to -12059	-5439 to -5380	ChrX:7151363-7151422	ChrX:7574237-7574296	15
ADS1183-FS	Proximal Promoter	-6750 to -6714	-71 to -35	ChrX:7156731-7156767	ChrX:7579605-7579641	5
ADS442-FS	Intron 1	-2555 to -2533	+4125 to +4147	ChrX:7160926-7160948	ChrX:7583800-7583822	2
ADS443-FS	Intron 1	-2405 to -2353	+4275 to +4327	ChrX:7161076-7161128	ChrX:7583950-7584002	3
ADS443-FS2	Intron 1	-2319 to -2287	+4361 to +4393	ChrX:7161162-7161194	ChrX:7584036-7584068	3
ADS568-FS1	Intron 1	-2369 to -2287	+4311 to +4393	ChrX:7161112-7161194	ChrX:7583986-7584068	5
ADS568-FS2	Intron 1	-2238 to -2207	+4442 to +4473	ChrX:7161243-7161274	ChrX:7584117-7584148	4
ADS779-RS1	Intron 1	-2075 to -2145	+4605 to +4535	ChrX:7161406-7161336	ChrX:7584280-7584210	2
ADS1184-FS1	3′ downstream	+23773 to +23784	+30452 to +30463	ChrX:7187253-7187264	ChrX:7610127-7610138	2
ADS1184-FS2	3′ downstream	+23802 to +23840	+30481 to +30519	ChrX:7187282-7187320	ChrX:7610156-7610194	5
ADS1184-FS3	3′ downstream	+23863 to +23905	+30542 to +30584	ChrX:7187343-7187385	ChrX:7610217-7610259	3
